# Primary Omental Leiomyosarcoma With Pulmonary Metastases: A Case Report of Surgical Management and Chemotherapy Response

**DOI:** 10.7759/cureus.78901

**Published:** 2025-02-12

**Authors:** Cielo S Silva-Ramos, Natalia M Barron-Cervantes, Erik F Gardner-Hilbert, Luis F Arias-Ruiz, Victor De la Puente Díaz de León, Alejandro Alfaro-Goldaracena

**Affiliations:** 1 General and Gastrointestinal Surgery Service, Fundación Clínica Medica Sur, Mexico City, MEX; 2 General and Gastrointestinal Surgery Service, Fundación Clínica Médica Sur, Mexico City, MEX; 3 Anatomic Pathology, Fundación Clínica Médica Sur, Mexico City, MEX; 4 Oncology, Fundación Clínica Médica Sur, Mexico City, MEX

**Keywords:** gemcitabine, leiomyosarcoma, omentectomy, omentum, peritonectomy, pulmonary metastases

## Abstract

Primary omental leiomyosarcoma is an extraordinarily rare tumor, with only a limited number of cases reported in the medical literature. Early surgical intervention appears to be a key determinant in the successful treatment of this malignancy, although more cases are needed to establish a standardized approach for optimal management. Leiomyosarcomas originating from the omentum present unique diagnostic challenges due to their rarity and often nonspecific clinical and radiologic findings. The complexities of diagnosing and managing this condition are compounded in rural areas where access to advanced diagnostic tools and specialists is limited. Leiomyosarcomas appear on abdominal computerized tomography (CT) as heterogeneous solid masses with cystic areas that enhance with contrast. While imaging can suggest the diagnosis, histopathology is required for confirmation. These tumors are highly aggressive, and complete surgical resection is the only definitive treatment, with resectability depending on peritoneal implants or metastasis. Other treatments, such as chemotherapy, radiation, and embolization, have variable success. Liver metastasis is the most common and a poor prognostic factor. Despite alternative therapies, surgery remains the best option for long-term survival. This case not only highlights the rarity and diagnostic challenges of omental leiomyosarcoma but also underscores the importance of timely referral to specialized centers. It further emphasizes the need for comprehensive diagnostic evaluations and personalized treatment plans tailored to the unique characteristics of each patient. Addressing these challenges through early intervention and targeted therapies is crucial to improving outcomes for patients with rare malignancies like omental leiomyosarcoma.

## Introduction

A leiomyosarcoma (LMS) is a rare type of malignant soft tissue tumor that derives from smooth muscle, mainly found in the pelvis and retroperitoneum, most commonly described in the uterus [1]. Leiomyosarcomas comprise approximately 5%-20% of all soft-tissue sarcomas [2]. Primary malignant tumors of the greater omentum are uncommon neoplasms, in contrast to metastatic carcinomas that commonly originate from the stomach or ovaries. The embryologic origin of these tumors is diverse due to the variety of tissues present in the omentum, including blood vessels, lymphatics, and fat. Leiomyosarcomas of the greater omentum are particularly rare, with less than 30 cases documented in the literature [3]. Given this extremely low number of documented cases, their exact incidence is difficult to determine. However, soft tissue sarcomas themselves account for less than 1% of all malignancies [4]. Percutaneous biopsy for omental tumors, including leiomyosarcomas, is not always essential and is often approached with caution. Biopsy should be performed in cases where the tumor is unresectable due to extensive metastasis or invasion so it can help guide non-surgical treatments and if there is diagnostic uncertainty, especially when differentiating between benign and malignant omental lesions. Percutaneous biopsy should be avoided in the rest of the cases because of the high risk of tumor seeding and due to its high potential for false negatives as omental tumors can be heterogeneous, meaning the biopsy might sample a non-representative area and lead to an inconclusive or misleading diagnosis [5].

Abdominal CT is the first-line imaging for detecting a heterogeneous, enhancing mass with cystic or necrotic areas; it assesses peritoneal implants and metastases, with a sensitivity of 80-95% and a specificity of 70-85%. Other imaging modalities available include magnetic resonance imaging (MRI) which is better for soft tissue contrast, detecting hemorrhage, necrosis, and differentiating LMS from other soft tissue tumors, also useful when CT findings are inconclusive. This study presents comparable sensitivity and specificity, with 85-97% and 75-90%, respectively [6]. However, due to its aggressive behaviour and high risk of metastatic progression, surgery remains the cornerstone of diagnostic and curative treatment [7]. The number of chemotherapy sessions for LMS of the greater omentum varies depending on several factors, including tumor stage, response to treatment, and the patient's overall health. Neoadjuvant chemotherapy is used in unresectable or borderline cases to shrink the tumor before surgery, adjuvant chemotherapy can be used if there is a high risk of recurrence and palliative chemotherapy is indicated for metastatic disease [8]. We present the case of a 68-year-old female patient who presented to the oncological outpatient clinic referred from a rural hospital with an oppressive abdominal pain and an abdominal computed tomography (CT) scan compatible with malignant characteristics in a first-level private surgical center in Mexico City. This case is presented in order to further expand the knowledge about omental leiomyosarcoma.

## Case presentation

A 68-year-old Latin-American female patient presented to the oncological outpatient clinic at Fundación Clínica Médica Sur in Mexico City after being referred from a rural hospital in Hidalgo, México due to the lack of diagnostic equipment and specialists who could finish addressing her case. The patient presented a persistent abdominal pain localized to the mesogastrium referred to as a 10/10 intensity, radiating throughout the abdomen and unresponsive to oral medication for analgesia. This abdominal pain was accompanied by chills, without quantifiable fever. Upon further questioning, the patient denied nausea or vomiting. Her relevant past medical history only included a history of insulin resistance of ten years of evolution without treatment managed only with diet and exercise. During her stay at the rural hospital, laboratory studies were requested, which revealed leukocytosis predominantly due to neutrophilia. The only image study that was performed was an abdominal CT scan, the patient was only able to share the final report which showed a 6.0 x 8.0 cm mass in the right hypochondrium, compatible with malignancy. No further description was added to the report. Upon her arrival to our oncological outpatient clinic, a complete physical examination, laboratories and complementary imaging studies were performed. On physical examination, the abdomen was soft, without evidence of palpable masses, and tender on deep palpation, with generalized pain but no signs of peritoneal irritation. Laboratory results still presented leukocytosis predominantly due to neutrophilia.

**Figure 1:**
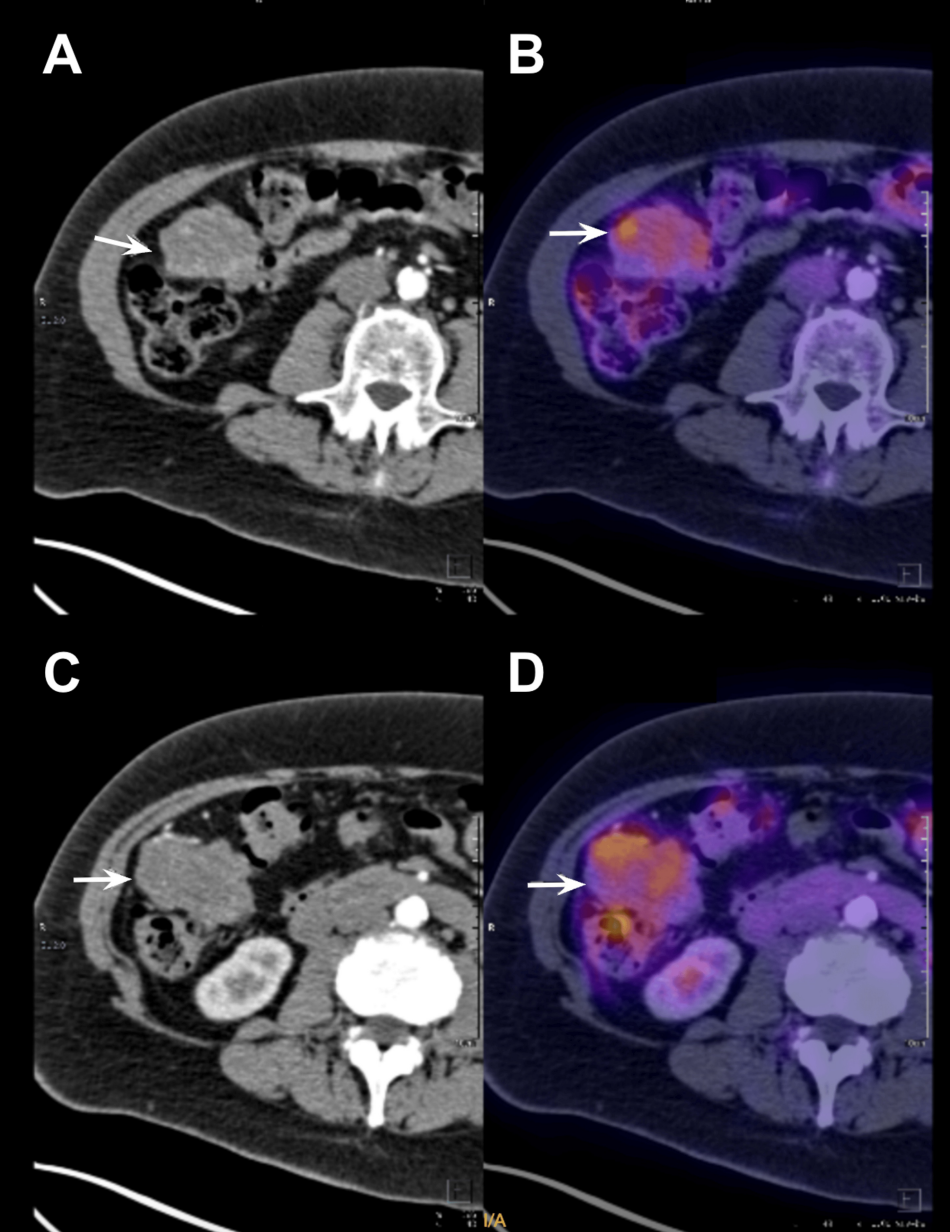
Preoperative PET-CT scan transverse view. Preoperative PET-CT scan presenting a solid peritoneal mass measuring 5.8 x 3.5 cm (white arrow) in the anterior right flank. A) Peritoneal mass (white arrow) adjacent to the ascending colon below the right kidney. B) Peritoneal mass (white arrow) exhibiting an average attenuation of 37 HU. C) Peritoneal mass (white arrow) adjacent to the ascending colon at the level of the lower pole of the right kidney. D) Peritoneal mass (white arrow) exhibiting an enhancement up to 60 HU in the venous phase.

**Figure 2:**
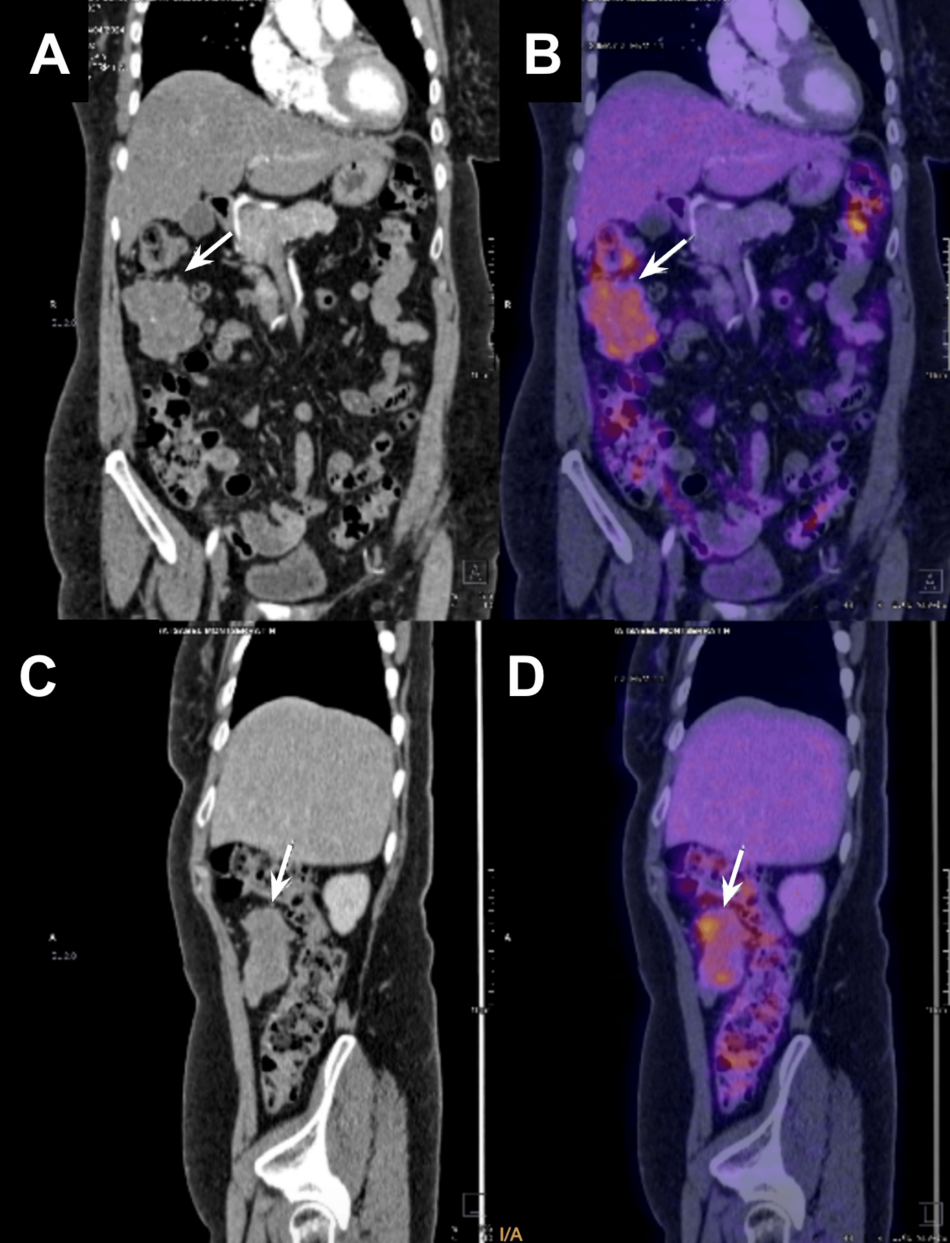
Preoperative PET-CT scan coronal and sagittal view. Preoperative PET-CT scan presenting a solid peritoneal mass measuring 5.8 x 3.5 cm (white arrow) in the anterior right flank. A) Coronal view of the peritoneal mass (white arrow) adjacent to the ascending colon below the right kidney. B) Coronal view of the peritoneal mass (white arrow) exhibiting an average attenuation of 37 HU. C) Sagittal view of the peritoneal mass (white arrow) adjacent to the ascending colon at the level of the lower pole of the right kidney. D) Sagittal view of the peritoneal mass (white arrow) exhibiting an enhancement up to 60 HU in the venous phase.

A positron emission tomography-computed tomography (PET-CT) scan was subsequently performed. This study revealed multiple solid nodules with a random distribution in the right lung, varying in size between 5 mm and 2 cm. The most significant nodule, located near the bifurcation of the segmental bronchi in the upper lobe, had an SUVmax of 6.2. Additionally, in the anterior right flank, adjacent to the ascending colon, a solid peritoneal mass measuring 5.8 x 3.5 cm with an approximate volume of 80 mL was observed. The lesion exhibited an average attenuation of 37 Hounsfield units (HU) in the non-contrast phase, it was hypervascular and heterogeneous in the arterial phase, and showed enhancement of up to 60 HU in the venous phase (Figure 1). The mass was in close contact with a proximal jejunal loop but maintained a fat interface with the colon. Heterogeneous radiotracer uptake was noted, with an SUVmax of 10.3 (Figure 2).

**Figure 3:**
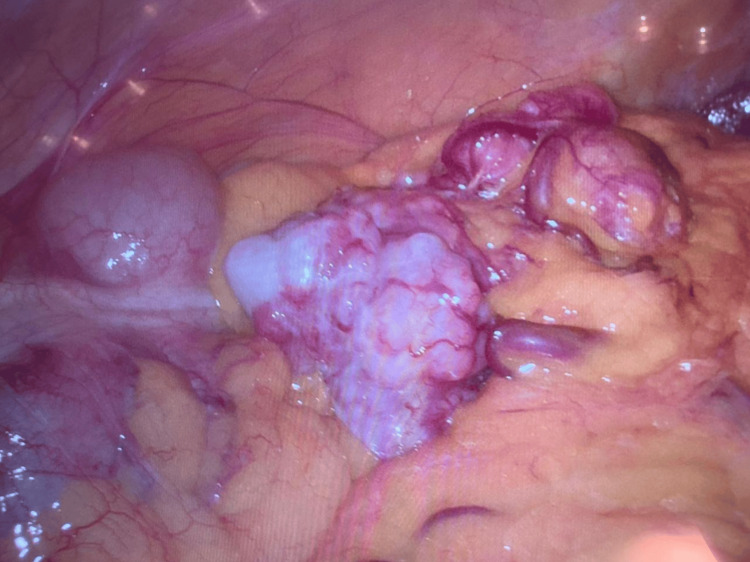
Laparoscopic view of the omental tumor. Laparoscopic direct view that shows a solid tumor arising from the greater omentum in the anterior flank.

**Figure 4:**
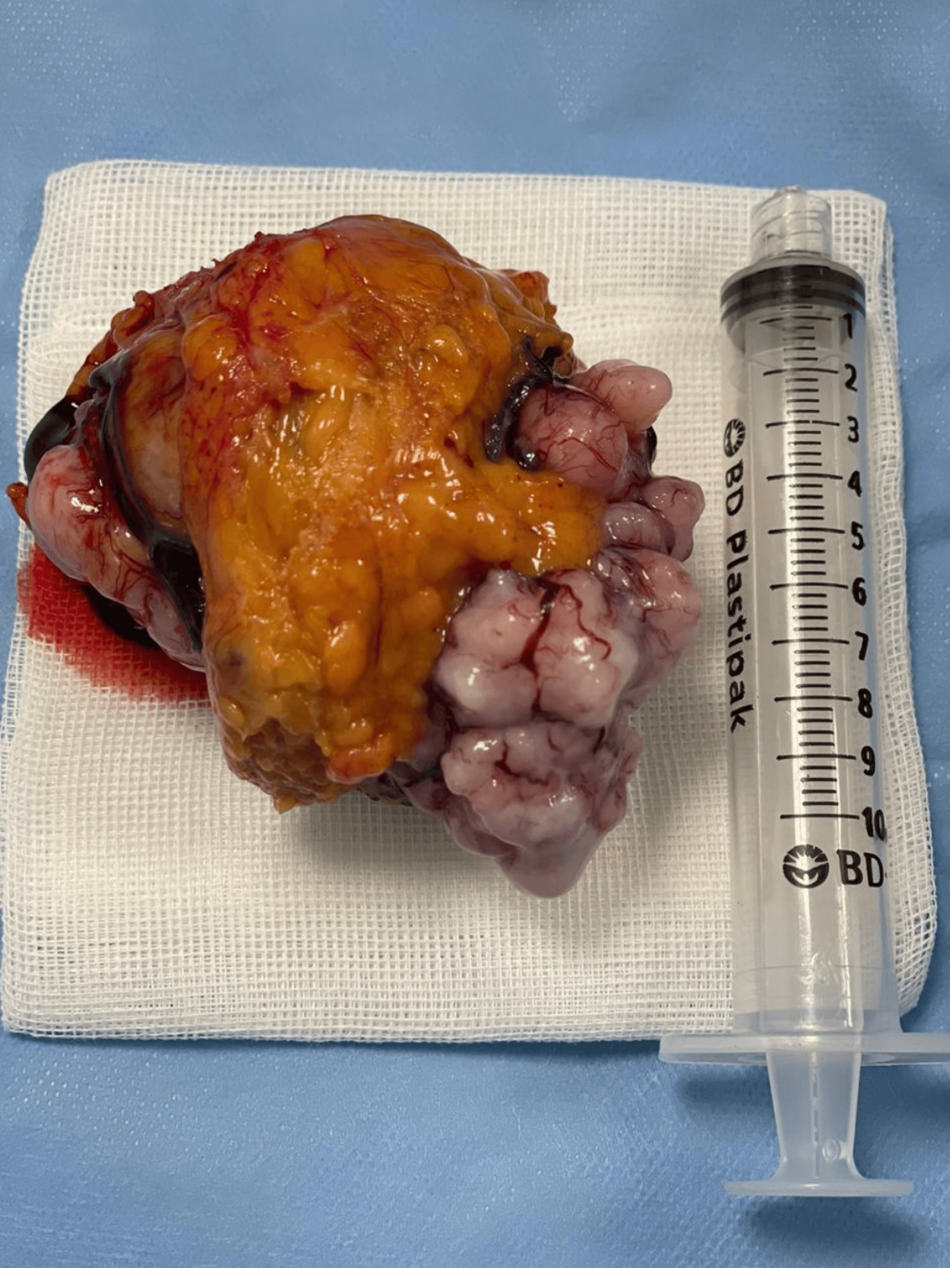
Macroscopic view of the tumor. Macroscopic view of the omental tumor besides a 10 mL syringe in order to show its approximate size of 8 cm.

The patient was referred for a surgical oncology consultation, where the decision was made to proceed with a completely laparoscopic approach, including diagnostic laparoscopy, abdominal tumor resection, partial peritonectomy of the right flank, and omentectomy. During the procedure, a laparoscope was introduced to evaluate the abdominal cavity, revealing an 8 cm mass originating from the greater omentum (Figure 3), along with an irregular nodule on the right flank, suggestive of implantation. The mass was excised and extracted through the umbilical port using a laparoscopic umbilical extraction bag. A partial peritonectomy was performed to remove the affected peritoneal tissue on the right side, and the omentum was resected to ensure complete removal of all visibly affected tissue. Notably, there was no conversion to open surgery, and no peritoneal irrigation was performed during the procedure. Both the tumor and the irregular nodule were sent to pathology for further histopathological evaluation (Figure 4).

**Figure 5:**
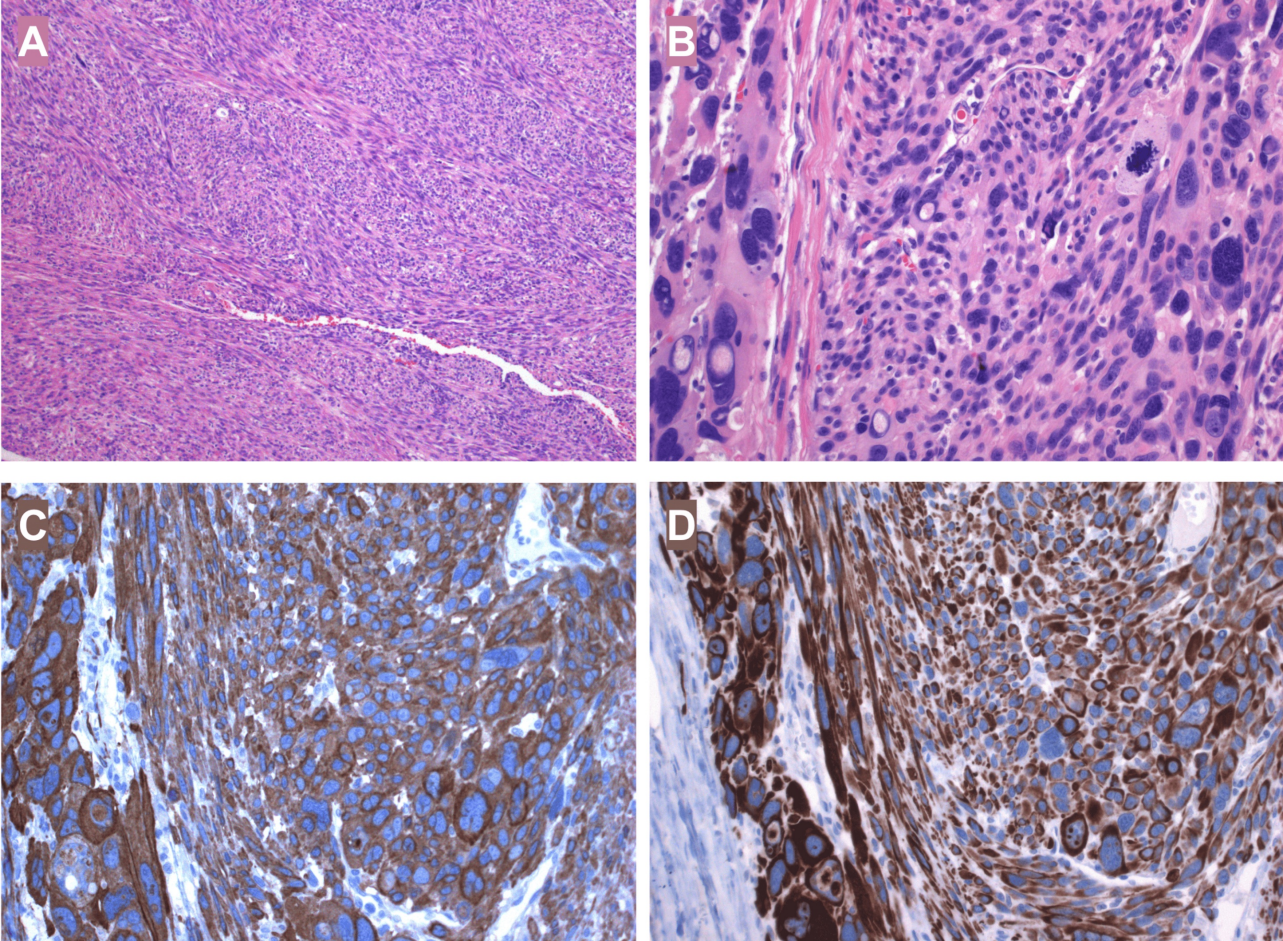
Histopathology of the tumor. A) On haematoxylin and eosin (H&E) sections the tumor exhibited intersecting fascicles of spindle cells with blunt-ended nuclei (40x magnification). B) Cells showed variation in size, prominent nuclear pleomorphism, vacuolated nuclei and abundant mitotic figures (200x magnification). C) Immunohistochemical analysis confirmed positivity for smooth muscle markers: smooth muscle actin. D) Immunohistochemical analysis confirmed positivity for desmin.

The specimen appeared grossly lobulated with a pink external surface. Upon sectioning, the cut surface was homogeneous, pale pink, and displayed a characteristic whirled pattern. Microscopic examination revealed intersecting fascicles of spindle cells with blunt-ended nuclei. The tumor exhibited prominent nuclear pleomorphism and a high mitotic activity, including atypical mitotic figures. Immunohistochemical analysis demonstrated positivity for smooth muscle actin and desmin, while CD117 was negative. The proliferation index, assessed with Ki-67 (MIB-1), was 15%. Based on these findings, a final diagnosis of leiomyosarcoma of the omentum was established (Figure 5).

Following surgery, the patient began treatment with liposomal doxorubicin, but experienced a severe anaphylactic reaction during the initial infusion, which required immediate discontinuation of the drug and administration of emergency supportive care. Due to the adverse reaction, the chemotherapy regimen was switched to gemcitabine, which was better tolerated. A follow-up PET-CT scan, performed after six treatment cycles, demonstrated stable disease with complete resolution of the right peritoneal mass. The pulmonary nodules showed no significant change in number or size, but there was a notable 48% reduction in metabolic activity, indicating a partial metabolic response to treatment. The patient tolerated gemcitabine well and reported improvement in symptoms, including reduced abdominal discomfort. She remains under active treatment and regular follow-up with medical oncology, with ongoing imaging to monitor disease status and ensure timely intervention if progression occurs. Follow-up consults were monthly for the first six months after finishing chemotherapy cycles, now, follow-up is every six months.

## Discussion

A leiomyosarcoma is a subtype of soft tissue sarcoma and they represent around 10-20% of all sarcomas. These malignant tumors commonly arise in the retroperitoneum, uterus, and extremities, and originate from smooth muscle cells or mesenchymal cell precursors [9]. Omental leiomyosarcomas, on the other hand, are exceptionally rare tumors with very few case reports available in surgical literature. The age of presentation has ranged from 26 to 85 years of age, with a median of 51.2 years of age as reported by Koga et al. in a list of documented cases of primary leiomyosarcoma of the greater omentum [10]. Tumors of the greater omentum usually present with early satiety, localized abdominal pain and vomiting, but they can also present differently as the greater omentum extends from the greater curvature of the stomach towards the pelvis anterior to the small intestine, before it folds back on itself to drape over the transverse colon [11]. Abdominal CT is commonly considered the first-line imaging modality for detecting heterogeneous masses with both enhancing and cystic or necrotic areas. It is particularly useful for assessing peritoneal implants and metastases, with reported sensitivity ranging from 80% to 95% and specificity between 70% and 85%. This makes it an essential tool in the initial evaluation of suspected abdominal tumors, providing critical information on tumor characteristics and potential spread. In addition to CT, MRI serves as another important imaging modality, particularly when more detailed soft tissue contrast is required. MRI is especially advantageous in identifying hemorrhage, necrosis, and distinguishing LMS from other soft tissue tumors, offering superior resolution for soft tissue differentiation. It becomes particularly valuable when CT findings are ambiguous or inconclusive. MRI has demonstrated comparable sensitivity and specificity to CT, with sensitivity ranging from 85% to 97% and specificity between 75% and 90%, making it an effective alternative or complementary imaging tool for the evaluation of abdominal masses and soft tissue abnormalities [7]. In this case, PET-CT was conducted prior to MRI due to its availability at the hospital during the patient's evaluation and because of a high clinical suspicion of malignancy. At the same time, it is important to emphasize that the final diagnosis will be achieved through histopathology.

As these tumors are highly aggressive, definitive treatment is only achieved by complete surgical resection. The resectability of the tumor will largely depend on the presence of peritoneal implants or distal metastasis. Other treatments that have been reported in the literature include chemotherapy, transcatheter arterial embolization (TAE), radiation, percutaneous ethanol (PEI) therapy, and microwave coagulation therapy (MCT) with varying degrees of success. The most frequently reported site of metastasis is the liver and this finding is considered one of the worst prognostic factors for this disease. Although many different treatments have been proposed and tested, surgical resection remains the best practice to achieve long-term survival rates [10, 11].

Histologically, leiomyosarcomas form fascicles of spindle cells with blunt-ended nuclei, which feature nuclear pleomorphism and atypical mitoses. Necrosis can also be seen [12]. An important differential diagnosis is the gastrointestinal stromal tumor (GIST), another malignant neoplasm, which can not only originate from the digestive tract but also the omentum, retroperitoneum and mesentery [13]. Differential diagnosis between leiomyosarcomas and GISTs may be difficult using only hematoxylin and eosin staining, thus, as seen in this patient, immunohistochemical evaluation of the specimen is crucial to distinguish between both entities [14]. Smooth muscle differentiation is determined by the positivity of at least two smooth muscle markers: smooth muscle actin (SMA), desmin, smooth muscle myosin, calponin or h-caldesmon. CD117 and DOG1 must be negative [15]. Immunohistochemically, GISTs test positive for CD117 (c-Kit, 95%), DOG1 and CD34 (70%). In most CD117-negative cases, the diagnosis of GIST may be confirmed by the positivity to DOG1. It is imperative to note that some GISTs may also express h-caldesmon (80%) and SMA (25%), which can be misleading. On the other hand, desmin is a helpful marker to distinguish between the two, as it is rarely expressed by GISTs (5%) [12-15].

The prognosis for omental leiomyosarcoma has largely been determined to be poor. Although few reports of this disease can be found in the literature, in approximately 27 reported cases, 11 cases died during follow-up [2, 16]. Complete surgical resection remains the most important prognostic factor for survival and may benefit certain patients with metastatic disease such as pulmonary metastasis with a low number of metastases and appearing after primary resection [17]. Further analysis of outcomes is required to determine best treatment practices, however, the rarity of these tumors makes improvements and analysis difficult. Personalized treatment strategies are the current standard of care for patients with omental leiomyosarcoma, surgery being the cornerstone of treatment. Additional case reports will aid in the further and better understanding of the disease, as there are still many unknowns about this rare tumor.

This case underscores the diagnostic and therapeutic complexities of managing primary omental leiomyosarcoma, a rare and challenging malignancy. The patient's presentation with severe, unrelenting abdominal pain, combined with imaging findings of a large peritoneal mass, highlighted the critical need for a comprehensive diagnostic approach, particularly in rural areas where diagnostic capabilities are limited. In this context, timely referral to a specialized center, along with advanced imaging techniques such as PET-CT, proved essential in reaching a definitive diagnosis. Surgical intervention, including diagnostic laparoscopy and resection of the omental mass, was crucial for both confirming the diagnosis and achieving a significant reduction in tumor burden. Histopathological analysis confirmed the diagnosis of omental leiomyosarcoma, an uncommon tumor that requires a tailored approach for treatment. Despite the initial challenge of an adverse reaction to liposomal doxorubicin, switching to gemcitabine allowed the patient to tolerate treatment and showed promising results, with a partial reduction in metabolic activity of the pulmonary nodules and stable disease overall.

## Conclusions

This case highlights the importance of personalized treatment strategies, multidisciplinary collaboration, and close monitoring in managing rare malignancies. The successful management of this patient also emphasizes the critical role of early detection, accurate diagnosis, and timely interventions in improving patient outcomes. While further research and additional case reports are needed to refine optimal treatment protocols and better understand the prognosis of omental leiomyosarcoma, this case contributes valuable insights into the clinical management of this rare and often underrecognized malignancy. Continued exploration and documentation of similar cases are essential for advancing knowledge and improving therapeutic options for patients with omental leiomyosarcoma.
